# Variations in Patients’ Overall Assessment of Their Health Across and Within Disease Groups Using the EQ-5D Questionnaire: Protocol for a Longitudinal Study in the Swedish National Quality Registers

**DOI:** 10.2196/27669

**Published:** 2021-08-27

**Authors:** Fitsum Sebsibe Teni, Ola Rolfson, Nancy Devlin, David Parkin, Emma Nauclér, Kristina Burström, The Swedish Quality Register (SWEQR) Study Group

**Affiliations:** 1 Health Outcomes and Economic Evaluation Research Group, Stockholm Centre for Healthcare Ethics Department of Learning, Informatics, Management and Ethics Karolinska Institutet Stockholm Sweden; 2 Department of Orthopaedics Institute of Clinical Sciences, Sahlgrenska Academy University of Gothenburg Gothenburg Sweden; 3 Swedish Hip Arthroplasty Register Gothenburg Sweden; 4 Centre for Health Policy University of Melbourne Melbourne Australia; 5 Office of Health Economics London United Kingdom; 6 City, University of London London United Kingdom; 7 Equity and Health Policy Research Group Department of Global Public Health Karolinska Institutet Stockholm Sweden; 8 Health Care Services, Region Stockholm Stockholm Sweden; 9 See Acknowledgments

**Keywords:** EQ-5D, EQ VAS, experience-based values, health-related quality of life (HRQoL), hypothetical values, patient values, Swedish National Quality Registers, health state valuation

## Abstract

**Background:**

EQ-5D is one of the most commonly used questionnaires to measure health-related quality of life. It is included in many of the Swedish National Quality Registers (NQRs). EQ-5D health states are usually summarized using “values” obtained from members of the general public, a majority of whom are healthy. However, an alternative, which remains to be studied in detail, is the potential to use patients’ self-reported overall health on the visual analog scale (VAS) as a means of capturing experience-based perspective.

**Objective:**

The aim of this study is to assess EQ VAS as a valuation method with an experience-based perspective through comparison of its performance across and within patient groups, and with that of the general population in Sweden.

**Methods:**

Data on nearly 700,000 patients from 12 NQRs covering a variety of diseases/conditions and nearly 50,000 individuals from the general population will be analyzed. The EQ-5D-3L data from the 12 registers and EQ-5D-5L data from 2 registers will be used in the analyses. Longitudinal studies of patient-reported outcomes among different patient groups will be conducted in the period from baseline to 1-year follow-up. Descriptive statistics and analyses comparing EQ-5D dimensions and observed self-assessed EQ VAS values across and within patient groups will be performed. Comparisons of the change in health state and observed EQ VAS values at 1-year follow-up will also be undertaken. Regression models will be used to assess whether EQ-5D dimensions predict observed EQ VAS values to investigate patient value sets in each patient group. These will be compared across the patient groups and with the existing Swedish experience-based VAS and time trade-off value sets obtained from the general population.

**Results:**

Data retrieval started in May 2019 and data of patients in the 12 NQRs and from the survey conducted among the general population have been retrieved. Data analysis is ongoing on the retrieved data.

**Conclusions:**

This research project will provide information on the differences across and within patient groups in terms of self-reported health status through EQ VAS and comparison with the general population. The findings of the study will contribute to the literature by exploring the potential of self-assessed EQ VAS values to develop value sets using an experience-based perspective.

**Trial Registration:**

ClinicalTrials.gov NCT04359628; https://clinicaltrials.gov/ct2/show/NCT04359628.

**International Registered Report Identifier (IRRID):**

DERR1-10.2196/27669

## Introduction

### Background

According to the US Food and Drug Administration, patient-reported outcomes (PROs) are defined as outcomes reported by patients without interpretation by anyone else [1]. They provide important information on outcomes that matter to patients [2], such as symptoms, functional outcomes, and health-related quality of life (HRQoL) [3,4]. PROs are increasingly used in health care [5,6] with applications in informing clinical practice and guidelines, informing health policy as well as supporting drug approval process among others [7]. In addition, PROs can be employed in different areas such as population surveillance, individual patient–clinician interaction, and research [8].

Patients provide information on standardized questionnaires termed patient-reported outcome measures (PROMs) [9], which are categorized into generic and disease/condition-specific PROMs. Generic PROMs enable comparisons across different patient groups and allow overall evaluation of care and quality of life. EQ-5D and the 36-item Short-Form (SF-36) are among the most common generic questionnaires. Condition-specific PROMs are used to assess outcomes specific to particular diseases/conditions from the perspective of patients [9,10].

The EQ-5D is a generic questionnaire used to measure HRQoL worldwide for a range of conditions and treatments [11]. It has a descriptive system (a set of questions in an HRQoL questionnaire encompassing different dimensions of health, the answers to which form a profile of an individual’s health) where respondents report their health in 5 dimensions (mobility, self-care, usual activities, pain/discomfort, anxiety/depression). Each of the 5 dimensions is measured with 1 question on the descriptive system of the EQ-5D questionnaire. EQ-5D contains a visual analog scale (EQ VAS) component for recording the respondent’s overall assessment of her/his health. There are 2 versions of the instrument for use in adults: one with 3 levels of severity (EQ-5D-3L) (1=no, 2=some/moderate, and 3=extreme problems/confined to bed/unable to), resulting in 243 (3^5^) unique health states. The other version with 5 levels of severity (EQ-5D-5L) (1=no, 2=slight, 3=moderate, 4=severe, and 5=extreme problems/unable to), resulting in 3125 (5^5^) unique health states. A health state is defined by combining the severity level from each of the 5 dimensions (e.g. for EQ-5D-3L health state 11223; no problem in the mobility [level 1] and self-care [level 1] dimensions, some problems with performing usual activities [level 2], moderate pain/discomfort [level 2], and extreme anxiety/depression [level 3]) [11].

An EQ-5D health state can be summarized into a single index (EQ-5D index) by applying a formula that attaches specific weights to each severity level in each dimension; this set of weights is termed a value set. The weights in a value set reflect the relative importance of the health dimensions and severity levels. The EQ-5D index enables the ranking of health states and can be used as the quality component in the adjustment of life years for calculation of quality-adjusted life years (QALYs) to be used in economic evaluation [12]. Commonly, EQ-5D indices are anchored at 1 (full health) and 0 (state as bad as being dead), with states considered worse than being dead given negative values [13].

A value set can be obtained using different health state valuation methods; for example, time trade-off (TTO), VAS, the standard gamble (SG) method, and the discrete choice experiments (DCEs) [12]. Choice-based health state valuation methods, which involve choosing between alternative scenarios, are crucial to produce health state utility used in the calculation of QALYs for use in cost-utility analyses. Such health state valuation methods are SG, TTO, and DCEs. SG is considered to have the strongest theoretical foundation on the basis of entailing the attributes of being choice based and incorporating an element of uncertainty. However, in terms of feasibility, TTO showed a better response rate [14] and is commonly employed in health state valuation. The VAS valuation method involves rating health states on the VAS scale. This leads to choice-based methods being preferred over it, as they are considered to allow choice/trade-off [15].

Currently, more than 30 countries have developed value sets for the EQ-5D-3L, predominantly using the TTO method. Some of the value sets have employed the VAS method, where respondents are asked to value described health states on the EQ-5D VAS. The EQ-5D VAS has a similar “thermometer”-like design as the EQ VAS, but when used for valuation purposes, respondents are given instructions to value a series of hypothetical EQ-5D health states by indicating where on the 0-100 line of the VAS they lie [12,13,16]. The EQ-5D-3L and EQ-5D-5L sample questionnaires containing the descriptive system and EQ VAS are presented in Multimedia Appendices 1 and 2 (Reproduced by permission of the EuroQol Research Foundation). In the development of value sets for the EQ-5D-5L, the TTO method is currently used together with the DCE method based on the EuroQol Valuation Technology (EQ-VT) protocol [17,18] for about 30 countries.

Based on the perspective taken by respondents, valuation of health states could be performed through a hypothetical or experience-based perspective. While value sets have usually been based on members of the general population’s values of health states described to them (hypothetical values), another approach involves individuals in the general population valuing their own current health state (experience-based values) [19-21]. There are also studies where patients valued hypothetical health states [22,23]. A large majority of the value sets have employed a hypothetical perspective [16,17]. Many valuation studies which used an experience-based perspective have also been conducted, including studies in Sweden [24-29]. According to the Dental and Pharmaceutical Benefits Agency in Sweden, the agency that determines state subsidization of a pharmaceutical product, experience-based values are given priority over hypothetical values [30,31]. There is a growing discussion and interest in experience-based perspective in health state valuation, based on general populations’ and patient populations’ valuation of health states globally as shown in different literature [19-21,32-41].

As a health state valuation method, the advantages and possible limitations of VAS have been discussed in comparison to other methods such as TTO and SG. The advantages include being quick to complete and relatively easy for self-administration [42]. However, the above discussed arguments of lack of theoretical basis and not being choice based have also been raised [42,43]. Besides, this idea has been challenged by questioning the need for valuation methods to be based on utility theory [44]. It was pointed out that empirical performance should be used to select the relevant valuation method and VAS valuation was regarded advantageous over other methods in this respect [44]. A recent paper discussed the issue of anchoring at “dead” (ie, to assign 0 to the state of being dead for the calculation of QALYs) and alternative approaches to remedy challenges associated with anchoring at “dead.” The different alternative approaches provided in the paper indicate the potential of VAS to be used in economic evaluation [45].

Some studies comparing VAS with TTO and SG reported the advantage of VAS in terms of feasibility, whereas in other studies it was shown that VAS values differ considerably from TTO and SG values [14,46-52]. Specifically, in terms of feasibility, that is, response rate and cost of administration, findings indicated that VAS performed better than the other methods [14,46-48]. However, correlations of VAS values with those based on the TTO and SG methods were low to moderate, leading the authors to raise concerns regarding the use of VAS in health state valuation [14]. VAS values have also been compared with results from the TTO and DCE methods among patients, professionals, and laypersons. The decision to use VAS or TTO for individual patients and TTO or DCE for laypersons was recommended based on previous findings [49]. Transformation of VAS values to SG and TTO values through power functions has also been explored [50,51]. Concerning this, advise against transformation between VAS values and SG values through power function was expressed due to a lack of theoretical relationships [52]. In short, the studies explored the relationship of VAS with other valuation methods such as TTO and SG, indicating differences in valuations.

EQ VAS, as a component of the EQ-5D instrument, has been used to derive experience-based VAS value sets by using individuals’ overall assessment of their health reported on the EQ VAS to summarize how good or bad the health state they report is. These value sets have been developed in countries such as Sweden, Germany, China, and Canada [24-28]. Applying such value sets, studies reporting population reference values (norms data) and those that compared problems reported on EQ-5D dimensions with EQ VAS values were conducted [53,54]. Furthermore, another study compared patient value sets with that of the general population [55]. In addition, comparisons involving experience-based values developed using patients’ own EQ VAS values across 15 countries indicated significant differences in valuations of the same health states [32]. Similarly, EQ VAS values provided to the same health states by patients with 4 different medical conditions were also found to be different [56]. The cited studies showed the development of value sets based on EQ VAS and their application in addressing different questions in HRQoL research.

EQ VAS as a component of the EQ-5D has been in routine use with its validity and reliability demonstrated in different studies. Specifically, the EQ-5D questionnaire is employed in several Swedish National Quality Registers (NQRs) [57], making it possible to investigate the relationship between EQ-5D health states and self-reported EQ VAS values in different patient populations. As to the routine performance of EQ VAS in clinical settings, its significance as a possible diagnostic tool to predict frailty, the feasibility of its inclusion in daily patient diaries, and its performance in the National Health Service in the United Kingdom have been reported [58-60]. The validity, reliability, and responsiveness of EQ VAS values have also been shown by studies in different countries, including Sweden, in the general population, and in specific patient groups [26,47,61-66].

As shown above, TTO has been employed commonly for health state valuation, while VAS has also been employed in several studies [12,16]. While hypothetical perspectives were used commonly [16], increasing interest in experience-based perspectives was shown [19-21]. VAS has demonstrated advantages over other valuation methods in terms of feasibility [14,46-48]. However, arguments for and against the potential of VAS for use in health states valuation have been forwarded [42-44]. Studies employing EQ VAS in the valuation of health states and in reporting health have been conducted [24-29,53-55]. However, in the context of patient valuations of their own health, there is a knowledge gap in the literature regarding the relationship between the EQ-5D health states and self-assessed EQ VAS values across and within patient populations.

This research project will contribute to addressing the literature gap by adding to the current literature and international debate on the role of EQ VAS as a valuation method for experience-based health states. Addressing this issue will be facilitated through investigating large data sets containing PRO records of different patient populations covering a wide variety of conditions within the 12 Swedish NQRs. Both the EQ-5D-3L and EQ-5D-5L health states will also be investigated.

### Objective

The research project aims to assess EQ VAS as a valuation method with an experience-based perspective through a comparison of its performance across and within patient groups, and with that of the general population in Sweden. The following research questions will be investigated:

- How do EQ-5D health states and self-assessed EQ VAS values vary across and within patient groups, and at baseline and 1-year follow-up, and in comparison to the general population data?
- To what extent do EQ-5D dimensions predict EQ VAS values, and how do the resulting experience-based patient value sets differ when estimated from patients’ data at baseline and 1-year follow-up and how do the EQ VAS values predicted from EQ-5D dimensions differ between different patient groups?
- How do these patient value sets modeled using data from the registers compare with the Swedish VAS and TTO experience-based EQ-5D value sets obtained from the general population?
- How do value sets for EQ-5D-3L, derived from EQ VAS, differ from value sets for EQ-5D-5L, derived from its EQ VAS?

## Methods

### Study Design

A longitudinal study involving analyses of data on different patient groups will be conducted by assessing PROs from baseline to 1-year follow-up. The data from patients will be compared with cross-sectional survey data from the general population.

### Data Sources

Data from 12 NQRs on about 700,000 patients with PRO records will be included from the over 1.4 million patients in the registers. Clinical data (age, sex, BMI, diagnosis/es, and interventions) and PROs data (EQ-5D-3L, and EQ-5D-5L) will be retrieved from the registers. Data from cross-sectional population surveys in Sweden will be included for comparison; about 45,000 records were used in developing the Swedish experience-based VAS and TTO value sets [26]. The registers included in the project are described in Tables 1 and 2.

**Table 1:** General information on the 12 National Quality Registers [57,67].

Register	Diagnosis/condition	Intervention^a^	Start year	Unique patients^b^	New entries per year^b^
Better management of patients with Osteoarthritis (BOA)	Hip, knee, hand osteoarthritis	Supported Osteoarthritis Self-Management Programme (SOASP)	2008	110,000	18,000
Swedish Ankle Registry (Swedankle)	Osteoarthritis and inflammatory conditions in the ankle	Total ankle arthroplasty, ankle arthrodesis procedures	1997	4000	400
Swedish National Anterior Cruciate Ligament Register (xBase)	Cruciate ligament injuries	Cruciate ligament surgery	2005	46,000	4000
Swedish Fracture Register (SFR)	All types of fractures including vertebral/spinal fractures	Surgical and nonsurgical treatments	2011	430,000	82,000
Swedish Heart Failure Registry (SwedeHF)	Chronic heart failure	Pharmacological treatment, physical activity	2003	92,000	9500
Swedish Hip Arthroplasty Register (SHAR)	Hip osteoarthritis and other hip joint diagnoses	Hip replacement	1979	370,000	25,000
Swedish Knee Arthroplasty Register (SKAR)	Knee osteoarthritis and other knee joint diagnoses	Knee replacement	1975	220,000	16,000
Swedish National Quality Register for Bipolar Disorder (BipoläR)	Bipolar affective disorder	Pharmacological treatment, patient education	2004	21,000	1500
Swedish National Registry for Respiratory Failure (Swedevox)	Respiratory failure	Long-term oxygen therapy	1987	20,000	1200
Swedish Rheumatology Quality Register (SRQ)	Rheumatic diseases	Medical treatment, rehabilitation	1995	80,000	7000
Swedish Spine Register (Swespine)	Spinal stenosis, disk hernia, and other spinal diagnoses	Spine surgery	1993	130,000	10,000
Swedish Registry for Systematic Psoriasis Treatment (PsoReg)	Psoriasis	Systemic treatment for psoriasis	2007	6500	500

^a^An intervention refers to surgeries or other forms of treatments provided to patients in the registers.

^b^Information on the number of patients and new entries per year was received from registers.

**Table 2:** EQ-5D data collected at the 12 National Quality Registers [57,67].

Register	Follow-up times
Better management of patients with Osteoarthritis (BOA)	First visit, 3 and 12 months; 1, 2, 3, 4, 5, 6, and 7 years after (100 patients per year are randomized to continued follow-ups)
Swedish Ankle Registry (Swedankle)	Before surgery, 6 months, 1 and 2 years after
Swedish National Anterior Cruciate Ligament Register (xBase)	Before surgery, 1, 2, 5, and 10 years after
Swedish Fracture Register (SFR)^a^	A week before injury (recall) and 1 year after
Swedish Heart Failure Registry (SwedeHF)	At new visit, within 6 months and 1 year, once every year
Swedish Hip Arthroplasty Register (SHAR)	Before surgery, 1, 6, and 10 years after
Swedish Knee Arthroplasty Register (SKAR)	Before surgery, 1 year after
Swedish National Quality Register for Bipolar Disorder (BipoläR)	At visit
Swedish National Registry for Respiratory Failure (Swedevox)	At treatment start, 1 year after
Swedish Rheumatology Quality Register (SRQ)	At visit
Swedish Spine Register (Swespine)	Before surgery, 1, 2, 5, and 10 years after
Swedish Registry for Systematic Psoriasis Treatment (PsoReg)	At each revisit due to psoriasis/visit to the skin clinic/telephone conversation with a dermatologist

^a^Baseline data in SFR are collected by a recall of a few weeks after the occurrence of fracture.

### Plan for Data Analyses

The analyses will focus on the 3 main data components coming from the EQ-5D instruments: data collected by the EQ-5D-3L (and EQ-5D-5L for some registers) descriptive systems on the 5 dimensions, the EQ VAS value self-assessed by patients, and indices resulting from transforming the EQ-5D-3L health states into a single index using the Swedish EQ-5D-3L experience-based VAS value sets.

The data from the NQRs will be pooled and diagnoses in each patient group will be used to identify the different subgroups. Records of patients with complete data on age, sex, diagnosis at baseline, and PROs at baseline and 1-year follow-up will be included in the analysis. Although different follow-up times are available in the registers as shown in Table 2, the baseline and 1-year follow-up data will be used in the comparison across the different patient groups. To make comparisons with findings from the patient data, demographic, BMI, and EQ-5D-3L data from the general population survey covering about 50,000 participants will be employed. Detailed information on the general population data is available elsewhere [26].

All analyses will be conducted using SAS version 9.4 (SAS Institute Inc.) and R version 3.6.2 (R Foundation for Statistical Computing).

### EQ-5D Health States and Observed EQ VAS Values

In addressing the first research question, the proportion of problems (no problem [level 1], some problems [level 2], and severe problems [level 3]) for each dimension and observed EQ VAS values of patients, at baseline and 1-year follow-up, will be compared across and within the patient groups (as well as subgroups based on diagnosis groups) and with the general population data. These descriptive analyses will also be presented by age groups, sex, BMI, and American Society of Anesthesiologists (ASA) physical status classification system (for patient groups with data on ASA class; Tables 1 and 2). Comparison of EQ VAS values by sex in the different patient groups, controlling for age, will be performed using analysis of covariance. Cluster analysis will be performed to assess the distribution of the EQ VAS value in the different patient groups at both baseline and 1-year follow-up.

Furthermore, pooled data from all NQRs will be used to analyze EQ VAS and to explore the influence of both patient characteristics and the patient group (registers). This will be performed by accounting for the grouping of patients by register. Linear mixed effects models with the patient group, preoperative EQ VAS (for the analysis at 1 year and the change from baseline), intervention type, age, and sex as fixed effects, and the patient group as the random effect will be used. Age and sex have been shown to influence EQ VAS values in previous studies [68,69].

Change over time in terms of the proportions of problems reported in each dimension will be presented using the Paretian Classification of Health Change, introduced to apply the Pareto Principle to EQ-5D health states [70]. This analysis will be performed by calculating the proportions of changes in health states from baseline to 1-year follow-up. The changes will be categorized as “no problem” (health state 11111 at both baseline and 1-year follow-up), “no change” (same health state at both baseline and 1-year follow-up), “improved” (improvement in at least one dimension without worsening in any other), “worsened” (worsening in at least one dimension without improvement in any other), and “mixed” (a mix of improvement and worsening) [70]. These changes will be analyzed descriptively in different patient groups and subgroups. In addition, changes in EQ VAS in the Paretian Classification of Health Change categories will be analyzed.

### Value Sets Based on Observed EQ VAS Values

In addressing the second research question, data on the EQ-5D health state and observed EQ VAS value at baseline and 1-year follow-up will be included in the respective analyses. To assess how well the observed EQ VAS value reflects the EQ-5D health states, the EQ-5D-3L dimensions will be analyzed as possible predictors of the observed EQ VAS value through regression models, such as ordinary least squares and generalized linear models, in each patient group and the pooled data. Both unadjusted and adjusted (for age and sex) models will be used. Corresponding analyses will be performed for EQ-5D-5L dimensions.

Based on results from predictive performance measures of the models, such as mean absolute error and root mean square error, the most appropriate models will be selected. These models will be used to develop patient value sets based on observed EQ VAS values assessed by the respective patient groups and the pooled data. In this process, value sets will be created for both baseline and 1-year postoperative follow-up.

### Comparison of Patient Value Sets With the Swedish Experience-Based VAS and TTO Value Sets

In addressing the third research question, value sets elicited for each patient group using EQ-5D-3L will then be compared with the Swedish experience-based VAS and TTO value sets for EQ-5D-3L, which are elicited from the general population [26]. This will be performed by comparing the regression coefficients (indicating the levels of decrement from full health) of the severity levels in each dimension across patient groups and the general population data. Furthermore, EQ-5D-3L indices calculated based on the value sets elicited from patients will be compared with those of the Swedish experience-based VAS (see model 4 in Table S4 of the supplementary material in Burström et al [26]) and TTO (see model 4 in Table 3 in Burström et al [26]) value sets to assess the levels of agreement. This will be performed using Lin’s concordance correlation coefficients or intraclass correlation coefficient [71].

### Comparison of EQ-5D-3L and EQ-5D-5L Value Sets From Patients in the BOA Register and Swedish Hip Arthroplasty Register

In addressing the fourth research question, a comparison of the value sets for the EQ-5D-3L and EQ-5D-5L versions modeled based on data from patients in the BOA and Swedish Hip Arthroplasty Register (SHAR) will be performed. The 2 patient groups were chosen because it is in these registers EQ-5D-5L data are available. Specifically, the comparisons will assess coefficients of the value sets for the 2 EQ-5D versions, EQ-5D index changes between adjacent health states, and the range of the indices (from minimum to maximum values in each index). Furthermore, analysis to compare the difference in EQ-5D index between comparable EQ-5D-3L and EQ-5D-5L health states will be performed through descriptive statistics using 243 health states from EQ-5D-3L and corresponding states from EQ-5D-5L. For a description of terms discussed in this paper, please see Textbox 1.

**Description of terms.**

**EQ-5D Dimensions**

In both EQ-5D-3L and EQ-5D-5L versions, the 5 domains (mobility, self-care, usual activities, pain/discomfort, and anxiety/depression) constituting the EQ-5D descriptive system [13].

**EQ Visual Analog Scale (VAS)**

A 20-cm vertical scale where respondents describe the overall rating of their health-related quality of life. It ranges from 0 (the worst imaginable health state) to 100 (the best imaginable health state) [13].

**Health State**

It is also described as an EQ-5D profile. It summarizes the severity of levels described in the 5 EQ-5D dimensions [13]. For example, health state 11111 describes no problems in all the 5 dimensions.

**Value Set**

An EQ-5D value set contains values for every possible EQ-5D health state. These values are calculated using algorithms providing weights to the level of problems reported on each EQ-5D dimension [13]. The algorithms are developed in valuation studies using different valuation methods.

**EQ-5D Index**

It is also known as EQ-5D value, score, or utility. It is a value that summarizes the value of a health state based on the set of weights assigned to the levels of severity for each dimension [13].

**Standard Gamble**

It measures preference under uncertainty. In this method, a respondent is presented with 2 alternatives. The comparison involves staying in a specific health state for a defined number of years (certain alternative) with that of a specific probability of being full health for the same period or a specific probability of immediate death (uncertain alternative) [72].

**Time Trade-off**

In time trade-off, respondents are asked to compare 2 certain alternatives. One alternative gives staying in a specific health state for a defined period. The alternative presents staying in full health for a specific duration, usually lower. The point at which the respondent becomes indifferent between the alternatives indicates his/her level of preference for the specific health state [72].

**Discrete Choice Experiment**

Respondents are presented with a choice between alternative hypothetical scenarios. The choices provided could vary across different levels of attributes or characteristics. Respondents choose from the alternative scenarios [73].

### Ethics Approval and Consent to Participate

The study has been approved by the Regional Ethical Review Board, Gothenburg (1185-18/2019-00812). General population survey data, with approval from the Regional Ethical Review Board, Stockholm (2020-03090), will be used for comparison with patient data sets. The data on the patients will be pseudonymized and stored at the Centre for Registers in Västra Götaland before access is provided to members of the research team authorized to do so. All analyses of the data will be on an aggregate level and there will be no individual-level reporting of data.

### Dissemination of Findings

Findings from studies in the project will be disseminated through publication in peer-reviewed scientific journals and presentations at national or international conferences.

## Results

The study project involves data from 12 NQRs and the general population. Data retrieval started in May 2019. Data of patients from the 12 NQRs and the survey conducted among the general population have been retrieved. Data analysis on the retrieved data is ongoing.

## Discussion

The project will provide information on the pattern of variation of EQ VAS value across patient groups and subgroups, and on how the pattern changes from baseline to 1-year follow-up. Information on the differences between experience-based values from patients and the general population in Sweden will also be provided. This could be an input to the discussion on the merits and characteristics of experience-based valuation.

This project is also expected to provide information on the level of importance of the different dimensions and levels of severity in the EQ-5D questionnaire to different patient groups. This will be assessed based on how a similar level of severity in one dimension (eg, pain/discomfort) is valued in different patient groups in terms of its impact on the EQ VAS value. Furthermore, the importance of different dimensions and severity levels to different patient groups will be assessed in comparison to the general population.

The project will also contribute to the discussion of valuation methods regarding the feasibility and appropriateness of EQ VAS as a valuation method. Based on the findings, the potential benefits of using experience-based EQ VAS values in clinical decisions—rather than values obtained from members of the general population valuing described health states—will be discussed. Furthermore, the potential role of value sets produced using EQ VAS values for use in resource allocation decisions will be discussed. If feasible value sets can be generated from patients’ self-assessed EQ VAS data, this not only provides a means of building patients’ views and experience into decision making, it also means not having to conduct separate, costly, and time-consuming stated preference studies.

## Appendix

Multimedia Appendix 1 Sample EQ-5D-3L questionnaire, English.

Multimedia Appendix 2 Sample EQ-5D-5L questionnaire, English.

## Abbreviations

ANOVA: analysis of variance
ASA: American Society of Anesthesiologists
BipoläR: Swedish National Quality Register for Bipolar Disorder
BOA: Better management of patients with Osteoarthritis
DCE: discrete choice experiment
EQ VAS: EuroQol visual analog scale
EQ-VT: EuroQol Valuation Technology
HRQoL: health-related quality of life
NQRs: National Quality Registers
PRO: patient-reported outcome
PROM: patient-reported outcome measure
PsoReg: Swedish Registry for Systematic Psoriasis Treatment
QALYs: quality-adjusted life year
SF-36: 36-item Short-Form
SG: standard gamble
SHAR: Swedish Hip Arthroplasty Register
SKAR: Swedish Knee Arthroplasty Register
SRQ: Swedish Rheumatology Quality Register
Swedankle: Swedish Ankle Registry
SwedeHF: Swedish Heart Failure Registry
Swedevox: Swedish National Registry for Respiratory Failure
Swespine: Swedish Spine Register
TTO: time trade-off
VAS: visual analog scale
xBase: Swedish National Anterior Cruciate Ligament Register
