# Supplementary material for: Variations in Patients’ Overall Assessment of Their Health Across and Within Disease Groups Using the EQ-5D Questionnaire: Protocol for a Longitudinal Study in the Swedish National Quality Registers
Source: JMIR Res Protoc. 2021 Aug 27;10(8):e27669. doi: 10.2196/27669 (PMC8433865; doi:10.2196/27669)
Supplement: Multimedia Appendix 1 [file resprot_v10i8e27669_app1.pdf]

Under each heading, please tick the ONE box that best describes your health TODAY.

MOBILITY

I have no problems in walking about ❑

I have some problems in walking about ❑

I am confined to bed ❑

SELF-CARE

I have no problems with self-care ❑

I have some problems washing or dressing myself ❑

I am unable to wash or dress myself ❑

USUAL ACTIVITIES *(e.g. work, study, housework, family or leisure activities)*

I have no problems with performing my usual activities ❑

I have some problems with performing my usual activities ❑

I am unable to perform my usual activities ❑

PAIN / DISCOMFORT

I have no pain or discomfort ❑

I have moderate pain or discomfort ❑

I have extreme pain or discomfort ❑

ANXIETY / DEPRESSION

I am not anxious or depressed ❑

I am moderately anxious or depressed ❑

I am extremely anxious or depressed ❑

Sample

© 1990 EuroQol Research Foundation. EQ-5D™ is a trade mark of the EuroQol Research Foundation. UK (English) v2.1

- We would like to know how good or bad your health is TODAY.
- This scale is numbered from 0 to 100.
- 100 means the <u>best</u> health you can imagine.
  0 means the <u>worst</u> health you can imagine.
- Please mark an X on the scale to indicate how your health is TODAY.
- Now, write the number you marked on the scale in the box below.

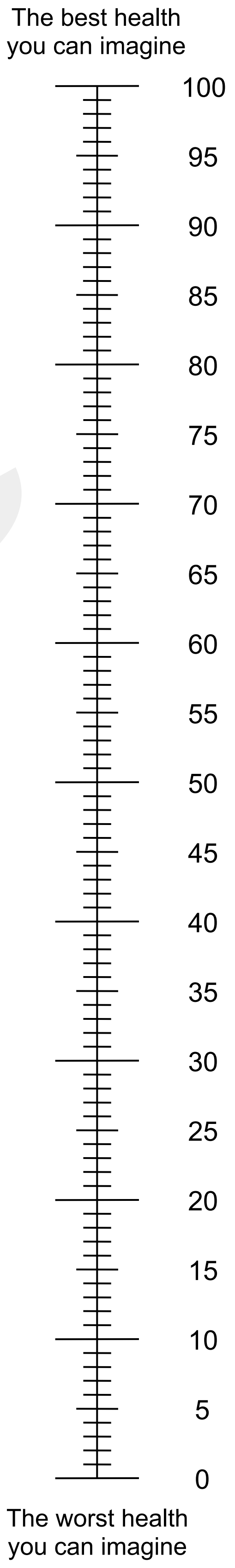

YOUR HEALTH TODAY = [ ]

Sample

© 1990 EuroQol Research Foundation. EQ-5D™ is a trade mark of the EuroQol Research Foundation. UK (English) v2.1
