# Supplementary material for: Variations in Patients’ Overall Assessment of Their Health Across and Within Disease Groups Using the EQ-5D Questionnaire: Protocol for a Longitudinal Study in the Swedish National Quality Registers
Source: JMIR Res Protoc. 2021 Aug 27;10(8):e27669. doi: 10.2196/27669 (PMC8433865; doi:10.2196/27669)
Supplement: Multimedia Appendix 2 [file resprot_v10i8e27669_app2.pdf]

Under each heading, please tick the ONE box that best describes your health TODAY.

MOBILITY

I have no problems in walking about ❑

I have slight problems in walking about ❑

I have moderate problems in walking about ❑

I have severe problems in walking about ❑

I am unable to walk about ❑

SELF-CARE

I have no problems washing or dressing myself ❑

I have slight problems washing or dressing myself ❑

I have moderate problems washing or dressing myself ❑

I have severe problems washing or dressing myself ❑

I am unable to wash or dress myself ❑

USUAL ACTIVITIES *(e.g. work, study, housework, family or leisure activities)*

I have no problems doing my usual activities ❑

I have slight problems doing my usual activities ❑

I have moderate problems doing my usual activities ❑

I have severe problems doing my usual activities ❑

I am unable to do my usual activities ❑

PAIN / DISCOMFORT

I have no pain or discomfort ❑

I have slight pain or discomfort ❑

I have moderate pain or discomfort ❑

I have severe pain or discomfort ❑

I have extreme pain or discomfort ❑

ANXIETY / DEPRESSION

I am not anxious or depressed ❑

I am slightly anxious or depressed ❑

I am moderately anxious or depressed ❑

I am severely anxious or depressed ❑

I am extremely anxious or depressed ❑

Sample

© 2009 EuroQol Research Foundation. EQ-5D™ is a trade mark of the EuroQol Research Foundation. UK (English) v1.2

- We would like to know how good or bad your health is TODAY.
- This scale is numbered from 0 to 100.
- 100 means the best health you can imagine.
  0 means the worst health you can imagine.
- Please mark an X on the scale to indicate how your health is TODAY.
- Now, write the number you marked on the scale in the box below.

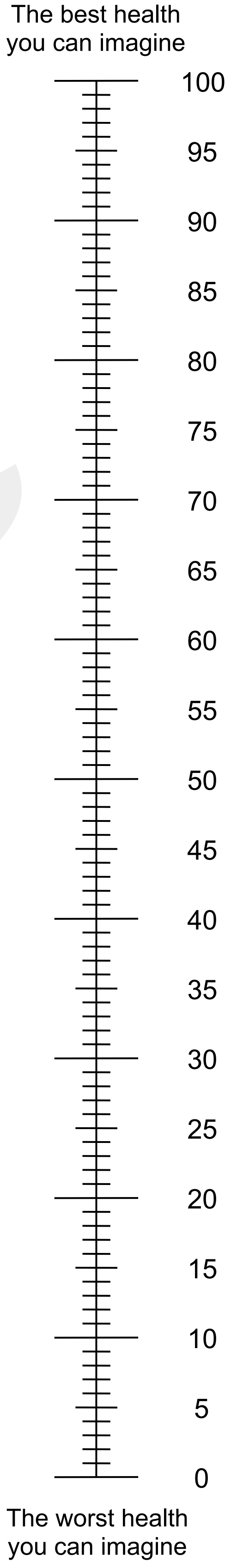

YOUR HEALTH TODAY = ☐

Sample

© 2009 EuroQol Research Foundation. EQ-5D™ is a trade mark of the EuroQol Research Foundation. UK (English) v1.2
